# Role of UPR^mt^ and mitochondrial dynamics in host immunity: it takes two to tango

**DOI:** 10.3389/fcimb.2023.1135203

**Published:** 2023-05-16

**Authors:** Manmohan Kumar, Shagun Sharma, Shibnath Mazumder

**Affiliations:** ^1^ Immunobiology Laboratory, Department of Zoology, University of Delhi, Delhi, India; ^2^ Faculty of Life Sciences and Biotechnology, South Asian University, Delhi, India

**Keywords:** UPR^mt^, mitochondrial dynamics, bacterial infection, ATFS-1, DRP1, MFN1, MFN2

## Abstract

The immune system of a host contains a group of heterogeneous cells with the prime aim of restraining pathogenic infection and maintaining homeostasis. Recent reports have proved that the various subtypes of immune cells exploit distinct metabolic programs for their functioning. Mitochondria are central signaling organelles regulating a range of cellular activities including metabolic reprogramming and immune homeostasis which eventually decree the immunological fate of the host under pathogenic stress. Emerging evidence suggests that following bacterial infection, innate immune cells undergo profound metabolic switching to restrain and countervail the bacterial pathogens, promote inflammation and restore tissue homeostasis. On the other hand, bacterial pathogens affect mitochondrial structure and functions to evade host immunity and influence their intracellular survival. Mitochondria employ several mechanisms to overcome bacterial stress of which mitochondrial UPR (UPR^mt^) and mitochondrial dynamics are critical. This review discusses the latest advances in our understanding of the immune functions of mitochondria against bacterial infection, particularly the mechanisms of mitochondrial UPR^mt^ and mitochondrial dynamics and their involvement in host immunity.

## Introduction

Host–pathogen interaction is an ever-emerging and evolving field. Almost every other day, pathogens pose new challenges, and the immune system provides respite and protection to the host. The innate immune system is the first line of host defense against invading pathogens (Medzhitov, 2010). Cells of the innate immune system (monocytes/macrophages, neutrophils, dendritic cells, etc.) are ubiquitous and recognize pathogens or their products with the help of germline-encoded pattern-recognition receptors (PRRs) triggering inflammatory responses, which help in pathogen clearance (Arango Duque and Descoteaux, 2014; Weiss and Schaible, 2015). Recent studies have reported that activation of innate immune cells is closely regulated by shifting in mitochondrial metabolism and physiology such as mitochondrial dynamics and mitochondrial proteastasis, and mitochondria provides a central scaffolding platform for innate immune signaling pathways induced by pattern recognition receptors (PRRs) in response to microbial ligands (Tur et al., 2017; Banoth and Cassel, 2018; Faas and De Vos, 2020).

Over the years, mitochondria were viewed as semi-autonomous cellular organelles required only for the bioenergetics and biosynthesis of macromolecules. Although the regulation of apoptotic signaling by mitochondria has been well appreciated during past years (Wang and Youle, 2009; Tiku et al., 2020), recent studies suggest that mitochondria also participate in several additional innate immune signaling pathways (Banoth and Cassel, 2018; Shen et al., 2022). Hence, mitochondria serve as the metabolic hubs and respond to intrinsic cues and environmental stressors with an implausible degree of plasticity, which enables them to participate in various cellular signaling pathways (Bahat and Gross, 2019; Chakrabarty and Chandel, 2021). However, the interplay between innate immunity and mitochondria goes well beyond the control of death or survival of the host cell. Recent pieces of evidence demonstrated that multitudinous innate immune signaling pathways are regulated by mitochondria, revealing mutual relationships between cellular metabolism and innate immunity. Besides functioning as an innate immune regulon, several studies have also documented the importance of this organelle in shaping adaptive immune responses, thus placing it at the crossroads of adaptive and innate immunity (Sena et al., 2013; Weinberg et al., 2015; Sandoval et al., 2018). Every single mitochondrion harbours trenchant agonists of inflammation (Nakahira et al., 2015), collectively known as danger-associated molecular patterns (DAMPs). Structural and functional damage to mitochondria due to bacterial infections leads to mitochondrial DAMPs causing inflammation, and even autoimmune neurodegenerative disorders. Recent studies have also established mitochondria as a key player in regulating and establishing immune responses against pathogens. Thus, production of anti-microbial proteins by immune cell survival against pathogenic infection depends on maintaining proper mitochondrial function by mitochondrial quality control (MQC). Among the different MQC mechanisms, mitochondrial unfolded protein response (UPR^mt^), mitophagy, biogenesis, and mitochondrial fusion-fission dynamics are essential. Bacterial infection induces mitochondrial stress which stimulates the mito-nuclear response pathway known as UPR^mt^ for counteracting mitochondrial stress and thus, maintains homeostasis. In addition, mitochondrial dynamics are also altered in response to bacterial infection, this could be a direct effect of virulent strategy adapted by bacterial pathogens or it could be a host cell response to restrain bacterial infection. However, the molecular mechanisms underlying both these events, and the dependency on each other still remains elusive. This review provides an updated perspective of UPR^mt^ and mitochondrial dynamics, the underlying molecular mechanisms, and how they conjure innate immunity to bacterial pathogens.

## The mitochondria-pathogen scrimmage

Mitochondrion plays a crucial role in regulating bacterial pathogenesis. Hence, bacterial pathogens have evolved strategies to subvert mitochondrial functions to promote proliferation and infection (Galmiche and Rassow, 2010; Escoll et al., 2016). For example, pathogenic bacteria manipulate mitochondria *via* the secretion of pore-forming toxins (Papatheodorou et al., 2006; Stavru et al., 2011; Palframan et al., 2012), manipulate mitochondrial-dependent cell death pathways *via* type III secretion system to promote their survival (Pallett et al., 2014; Arizmendi et al., 2016), produce electron chain transport inhibitors (Raveh et al., 2013), and iron chelators that perturb mitochondrial functions thereby aiding their survival (Kirienko et al., 2015).

Conversely, mitochondria have evolved several mechanisms to resist bacterial infections, like production of anti-bacterial ROS (West et al., 2011; Kumar et al., 2022b; Kumar et al., 2022a), inflammasome activation (Zhou et al., 2011), xenophagy (Gatica et al., 2018) and consequently apoptosis of infected cells (Deo et al., 2020; Kumar et al., 2022b; Kumar et al., 2022a). To achieve this, immune cells synthesize several factors such as antimicrobial peptides, cytoplasmic and cell-surface surveillance proteins, signaling proteins, and inflammatory cytokines to countervail bacterial infection; hence, proteostasis remains a challenge for immune cells under bacterial assault. Proteostasis depends on the subtle harmony between maintaining protein conformation, refolding of misfolded proteins, and degrading damaged proteins; the process is precisely regulated in the endoplasmic reticulum (ER). The ER and mitochondria are tightly associated with dynamic modules termed mitochondria-associated membranes (MAMs) or mitochondria-ER contact sites (MERCs), which provide an excellent platform for cross-talk between the two organelles (Missiroli et al., 2018; Namgaladze et al., 2019).

Following bacterial infection, damaged or misfolded proteins accumulate in the ER. Recent studies suggest that MAMs/MERCs act as hotspots through which misfolded proteins transit into mitochondria, implicating the role of this organelle in sequestering anomalous proteins, thereby restoring ER functioning (Li et al., 2019; Ruan et al., 2020). The build-up of such proteins in mitochondria triggers proteotoxic stress, thereby activating its protein quality control mechanism, known as mitochondrial unfolded protein response (UPR^mt^), as a proteostatic mechanism (Haynes and Ron, 2010; Jovaisaite et al., 2014; Tran and Van Aken, 2020).

## UPR^mt^: the mitochondrial SOS response

UPR^mt^ is an evolutionarily conserved response that enhances the transcription of protective factors and chaperones, which are involved in the homeostasis and repair of damaged or stressed mitochondria (Ryan and Hoogenraad, 2007; Quirós et al., 2016; Topf et al., 2016). Thus, besides playing a role in maintaining the mitochondria status quo, UPR^mt^ also aids in animal fitness and host survival under different conditions of stress such as oxidative stress, and proteotoxic stress. Notably, less is known regarding the regulation of the UPR^mt^ in vertebrates, and further studies are needed to understand this. Impaired protein import in the mitochondria has been linked with the activation of UPR^mt^ (Haastrup et al., 2023). And the import of mitochondrial protein is a biologist’s enigma! Around 99% of mitochondrial proteins are encoded in the nuclear genome. The proteins destined for mitochondria contain a signal sequence (or pre-sequences) that direct them to mitochondria from the cytosol. Mitochondrial matrix-targeting sequences are rich in hydrophobic amino acids, positively-charged basic amino acids (arginine and lysine), and hydroxylated amino acids (threonine and serine) and lack negatively-charged acidic residues (aspartate and glutamate). The amphipathic nature and positive charge of the pre-sequences is recognized by protein import receptors at mitochondria rather than a precise amino acid sequence for the translocation of the protein into the mitochondria. Thus, the postal addresses for mitochondrial proteins are ill-defined in terms of amino acid sequence or specific chemical moieties. It has been suggested that physio-chemical traits like pre-sequences’ ability to bind to specific receptors coupled with maintaining thermodynamic equilibrium favouring unfolded proteins facilitate mitochondrial import of the pre-sequences. Mitochondrial precursor proteins aren’t imported into mitochondria in their native state; they are unfolded during translocation and move across the import machinery as linear chains. The internal diameter of TOM (translocase of the outer mitochondrial membrane) is ~22 Å (Schwartz et al., 1999); hence, only small folded proteins can translocate. TIM (translocase of the inner mitochondrial membrane) contains a much narrower channel, and a slight steric hindrance halts translocation into the matrix, suggesting precursor proteins must be unfolded to pass through this channel. The overall mechanisms of mitochondrial import differ depending upon the charge, size, and presence of cysteine moieties (Harbauer et al., 2014), but grossly it has been suggested that the negative charge of the inner mitochondrial membrane attracts positively-charged mitochondrial targeting sequence (MTS) and the difference in membrane potential subsequently drives the positively charged pre-sequence across the inner membrane (Martin et al., 1991; Harbauer et al., 2014). The TOM complex serves as the entry point for most precursors at the outer membrane, and the binding of pre-sequences with TOM is facilitated by hydrophobic interactions (Genge and Mokranjac, 2022). TIM complex facilitates the entry of proteins into the mitochondrial matrix. The interaction between intermembrane space (IMS)-domains of TOM and TIM family members with the pre-sequence of the incoming precursor proteins facilitate the transfer of these proteins across the mitochondrial membrane.

## UPR^mt^ in *C. elegans*

**Figure 1:**
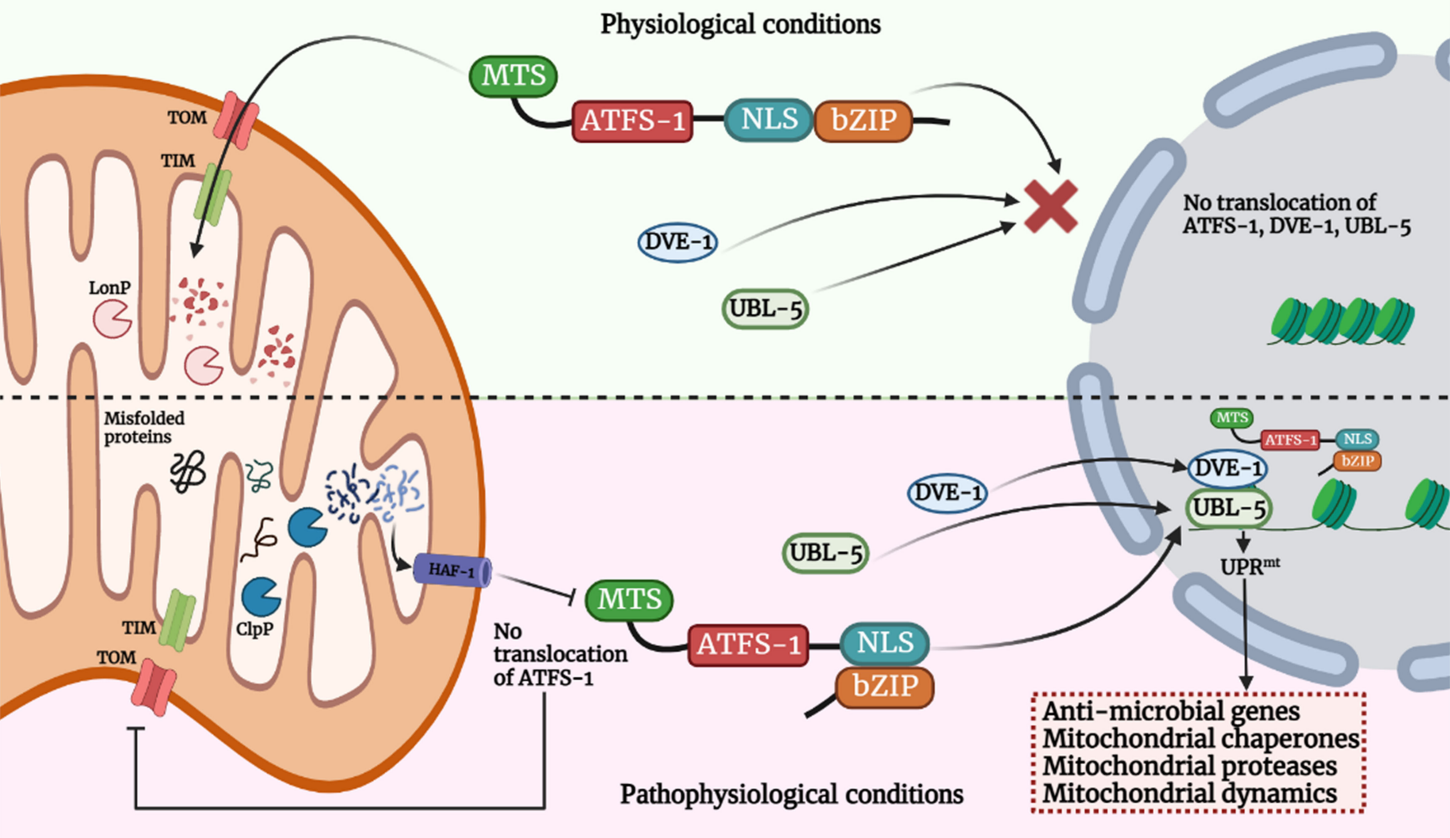
Model for UPR^mt^ signaling pathway. Activation of the UPR^mt^ in cells occurs in response to different stress signals such as mtROS, mutation in mtDNA, accumulation of misfolded mitochondrial proteins in the mitochondrial matrix or in IMS and alteration in mitochondrial membrane potential due to bacterial infection. Such events trigger the translocation of ATFS-1 (mammal homologue - ATF-5) which is bZIP protein, and contains MTS and NLS sequence into the nucleus and along with DVE-1 and UBL-5, it induces a transcriptional up-regulation program known as UPR^mt^. Under physiological conditions, it is imported in the mitochondria *via* TOM/TIM machinery and digested in the mitochondrial matrix by mitochondrial protease LonP which prevents activation of UPR^mt^. Under pathophysiological or stressed conditions, import of ATFS-1 is blocked in mitochondria. The misfolded proteins digested in the mitochondrial matrix *via* ClpP and efflux of such peptides into the cytoplasm *via* HAF-1 prevents ATFS-1 translocation into mitochondria, and therefore it moves into the nucleus and induces multiple genes which functions in restoring mitochondrial functions, innate immunity facilitating bacterial clearance. The image was created with the help of BioRender.com.

Activating transcription factor associated with stress (ATFS-1), a basic leucine zipper (bZIP) protein, contains both nuclear localization sequence (NLS) and MTS enabling this transcription factor to shuttle between the two organelles and helping mitochondria in establishing communication with the nucleus (Haynes et al., 2010; Nargund et al., 2012) thereby regulating UPR^mt^. The import of ATFS-1 into the mitochondrial matrix requires several factors, including the members of TOM/TIM complex, ETC, matrix-localized molecular chaperone mtHsp70 (Yoneda et al., 2004; Chacinska et al., 2009; Nargund et al., 2012; Rolland et al., 2019). Under normal conditions, ATFS-1 is imported into mitochondria due to the positively-charged MTS, where it is cleaved, and the protein is degraded by the matrix protease, Lon Protease (LONP) (Melber and Haynes, 2018). Under stress, mitochondrial import is compromised, and ATFS-1 undergoes a topological change and accumulates in the cytosol. Due to the presence of NLS, it translocates into the nucleus, functions with homeobox protein defective proventriculus homolog protein (DVE-1) and ubiquitin-like protein 5 (UBL-5), initiating the transcription of UPR^mt^ genes essential for a myriad of biological functions such as anti-microbial genes, protein folding machinery encoding genes, proteins regulating mitochondrial dynamics and mitochondrial proteases etc (Benedetti et al., 2006; Haynes et al., 2007; Nargund et al., 2012; Tian et al., 2016). (**Figure 1**). The MTS of ATFS-1 is relatively weak as compared to other mitochondrial proteins (Rolland et al., 2019; Shpilka et al., 2021; Xin et al., 2022) which renders ATFS-1 much more sensitive to the mitochondrial perturbations that in turn reduces import of ATFS-1 into the mitochondria. Thus, impairment in ATFS-1 translocation into the mitochondria serves as a rheostat for UPR^mt^ activation. Work emanating from several laboratories has identified that multiple components are required for UPR^mt^ activation, including sensors of mitochondrial dysfunction, regulators of mitochondrial-nuclear communication, chromatin regulators, and transcription factors collectively suggesting the dynamism in the regulatory mechanisms (Tian et al., 2016; Wang et al., 2022). It was proposed that a mitochondrial protease, ClpP-1, hydrolyses mitochondrial matrix proteins and generates peptides that might be exported into the cytoplasm through HAF-1, a transporter located in the inner membrane of mitochondria (Haynes et al., 2010). CLPP-1 is a protease that digest soluble proteins in an ATP-dependent manner in combination with another enzyme, AAA+ ATPase (Kang et al., 2002; Gottesman, 2003). CLPP-1 is localized at the mitochondrial matrix which implicates a role for CLPP-1-mediated proteolysis in signaling a proximal step in UPR^mt^ activation (Haynes et al., 2007). It has been suggested that the UPR^mt^ regulator HAF-1 plays a role in the mitochondrial import of ATFS-1 (Nargund et al., 2012). CLPP-1 digests proteins into small peptides which are then transported to cytoplasm *via* ABC transporter (HAF-1), and these peptides are processed into amino acids *via* cytoplasmic peptidases in cytoplasm. HAF-1 is a 677 amino acid protein with a putative N-terminal mitochondrial import signal, a transmembrane region and a single ATP-binding cassette. Deletion of mitochondrial import signal or ATP-binding cassette of HAF-1 results in the impairment in UPR^mt^ activation (Haynes et al., 2010). Importantly, contribution of HAF-1 in peptide efflux from stressed mitochondria is integrated with the prerequisite for CLPP-1-mediated proteolysis in the UPR^mt^. Together, these findings suggest efflux of peptides derived from stress-induced proteolysis is important to signaling the UPR^mt^. Most studies have been conducted in *Caenorhabditis elegans* where it has been observed that ATFS-1 is the essential protein responsible for UPR^mt^ activation. It was previously reported that GCN-2 (general control nonderepressible 2, a serine/threonine-kinase) phosphorylates translation initiation factor eIF2α upon mitochondrial stress in *C. elegans*, leading to the inhibition of global translation and restoration of mitochondrial homeostasis (Baker et al., 2012). But recently, a molecular mechanism has been reported (Li et al., 2022) for the activation of ATFS-1 under mitochondrial dysfunction. Mitochondrial stress induces vacuolar H^+^-ATPase (v-ATPase)/Rhe-dependent activation of TORC1 (target of rapamycin complex 1), which enhances translation of ATFS-1 protein (Shpilka et al., 2021; Li et al., 2022), and also reported that this pathway is independent of GCN-2-mediated signaling pathway, and contradicting the previous report. Moreover, recent study suggested several other organelles, such as lysosomes and ribosomes, are also involved in UPR^mt^ activation (Li et al., 2022). v-ATPase (vacuolar-ATPase) is a large, multi-subunit complex, ATP-dependent proton pumps that function in the acidification of intracellular compartments such as lysosomes (Forgac, 2007), and in addition, it has also been reported in the fusion of endosomal membrane (Peters et al., 2001). Knock-down of multiple subunits of v-ATPase (such as *vha-1*, *vha-4*, *vha-6*, *vha-10*, *vha-12*, *vha-15*, *vha-16*, *vha-19*) attenuated *cco-1* (cytochrome c oxidase subunit 1) or *mrps-5* (mitochondrial ribosomal protein S5) RNAi-induced UPR^mt^ activation in *C. elegans* (Li et al., 2022). Specifically, inhibition of *vha-1* significantly blocked transcription of several UPR^mt^ genes (e.g., *hsp-60*, *hsp-6*, *clec-4*, *gpd-2*) in response to *mrps-5* or *cco-1* knockdown (Li et al., 2022). And surprisingly, silencing of *vha-1*, *vha-4*, *vha-16*, *vha-19* didn’t have affect activation of ER-stress or cytosolic UPR. v-ATPase also acts as a crucial mediator in mTORC1 signaling pathway (Li et al., 2022). Pharmacological inhibition of TORC1 by Torin1, or silencing of *let-363* (mTOR) and *rheb-1* (upstream activator of TORC1) attenuated UPR^mt^ activation induced *via mrps-5* or *cco-1* knockdown, similar to the effects observed in case of v-ATPase RNAi (Li et al., 2022). Consistent with the importance of lysosomes in mTORC1 activation (Lawrence and Zoncu, 2019), inhibition of lysosomal acidification attenuated induction of UPR^mt^ genes, TORC1 activity (Fedele and Proud, 2020; Li et al., 2022) and accumulation of ATFS-1 (Li et al., 2022) which implicates the importance of lysosomes is essential for TORC1 and UPR^mt^ activation.

Furthermore, knockdown of ribosomal subunits, including large subunits (*rpl-14*, *rpl-25.1*, *rpl-27*, *rpl-36* and *rpl-43*) and small subunits (*rps-8* and *rps-10*) individually blocked *cco-1* or *mrps-5* RNAi-induced UPR^mt^ activation (Li et al., 2022). In addition, significant increase in polysomal mRNA of ATFS-1 was reported in response to *cco-1* RNAi, which was attenuated with *vha-1* RNAi co-treatment (Li et al., 2022). Knockdown of *cco-1* resulted in shifting from polysomes to monosomes, which confirms reduction in cytosolic translation in response to mitochondrial stress (D’Amico et al., 2017; Suhm et al., 2018; Molenaars et al., 2020). Together, these results suggest that increased translation of ATFS-1, mediated by v-ATPase/TORC1 and cytosolic ribosomes, is a key mechanism that leads to the accumulation of ATFS-1 protein for UPR^mt^ activation in response to mitochondrial stress.

Under stressed conditions, mitochondria enhance TORC1 activity *via* v-ATPase- and Rheb-dependent mechanism. TORC1 has been documented to be essential for UPR^mt^ activity (Shpilka et al., 2021), and activated TORC1 is associated with increased translation of ATFS-1, reliant on the cytosolic ribosomes (Li et al., 2022). Although, how TORC1 is activated in response to mitochondrial stress remains a mystery and highlights an important direction for future work. One possibility could be that the unfolded proteins produced upon mitochondrial stress might be transported from mitochondria to lysosomes, and eventually digested to peptides/amino acids within the lysosomes, which could then lead to TORC1 activation at the lysosomal surface (Zoncu et al., 2011; Shimobayashi and Hall, 2014; Wolfson and Sabatini, 2017; Lawrence and Zoncu, 2019), however, what type of peptides/amino acids led to TORC1 activation still needs an investigation. Thus, mitochondrial stress likely signifies an inimitable intrinsic cue for TORC1 activation through lysosome-derived peptides/amino acids (Hesketh et al., 2020); furthermore, stressed mitochondria might also establish direct contact with lysosomes *via* mitochondria–lysosome membrane contact sites (Wong et al., 2019). As v-ATPase are involved in fusion of endosomal membranes, hence, it facilitates transportation of mitochondria-derived unfolded proteins to lysosomes, and thus, are crucial in UPR^mt^ activation.

Under pathophysiological conditions, the molecular mechanism that exposes the NLS region of ATFS-1 in the cytosol is not well understood and merits further studies. Based on the studies concerned with functional alteration in ATFS-1 (Nargund et al., 2012; Rolland et al., 2019), it can be suggested that import of ATFS-1 is impaired due to reduction in mitochondrial membrane potential and hence, translocate into the nucleus; furthermore, another hypothetical explanation could be that ATFS-1 senses stress-induced structural-functional alterations of the translocases and other constituents of mitochondrial protein import complex, and translocate to the nucleus. Identifying bacterial virulence proteins-induced alterations in mitochondria that selectively inhibits mitochondrial import of ATFS-1 is vital to understand mitochondrial dysfunction in bacterial infections. Importantly, ATFS-1 per se regulates the induction of several core components of mitochondrial protein import complex core components (Chacinska et al., 2009), suggesting the cross-talk between ATFS-1 and mitochondrial protein import complex essential for the maintenance of mitochondrial health; the failure to import ATFS-1 provides a feedback loop to the nucleus to initiate mitochondrial protein import and restore normalcy.

Besides pathogenic insult, various other factors, such as impairment of ETC, alteration of mitochondrial network dynamics, deletion of mitochondrial DNA (mtDNA), inhibition of mitochondrial chaperones, impaired expression of genes regulating mitochondrial functioning like protein import, OXPHOS, coenzyme and lipid biogenesis also trigger UPR^mt^ in *C. elegans* (Nargund et al., 2012; Runkel et al., 2013; Qureshi et al., 2017; Voth and Jakob, 2017) which suggests the pluralism behind triggering UPR^mt^ and its impact on different physiological processes and pathologic manifestations.

## UPR^mt^ in higher vertebrates

UPR^mt^ is less well understood in other higher animals and mammals. It has been observed that besides pathogenic stress, other factors like the perturbation of mitochondrial ribosomes, ectopic expression of mutant ornithine transcarbamylase (OTC), and depletion of mtRNA induce UPR^mt^ in mammalian cells (Zhao et al., 2002; Wu et al., 2014; Moullan et al., 2015). Even the presence of cytosolic protein aggregates has been found to initiate UPR^mt^ in several neurodegenerative diseases (Zhu et al., 2021); however, the link between cytosolic, mitochondrial proteostasis and UPR^mt^ is unclear under such settings. In mammals, several orthologous transcription factors (ATF4, ATF5, CHOP and C/EBP-β) have been reported (Fiorese et al., 2016; Quirós et al., 2017; Melber and Haynes, 2018). It has been suggested that both ATF4 and ATF5 are involved in expressing stress-responsive and cytoprotective genes in mammals (Fiorese et al., 2016; Quirós et al., 2017), but their relationship is not known. Additionally, mammalian cells, on sensing dysfunctional mitochondria activate MEK/JNK2 pathway inducing downstream expression of CHOP and C/EBP-β, which in turn promotes the transcription of downstream stress-responsive genes (Wang et al., 2018). Further, it has been noted that CHOP regulate ATF5 expression to upregulate UPR^mt^ genes (Teske et al., 2013; Fiorese et al., 2016) by interacting with SatB2 (mammalian orthologue of DVE-1) and Ubl5 (mammalian orthologue of UBL-5) (Haynes et al., 2007). There are also reports implicating ATF4 plays an indirect role in inducing UPR^mt^ responses by mounting the general cytoprotective integrated stress response (ISR) (Quirós et al., 2017). Furthermore, another type of UPR^mt^ that occurs in the IMS has also been reported in mammals (Papa and Germain, 2011). IMS-UPR^mt^ instigates reactive oxygen species (ROS) production and triggers the phosphorylation of AKT kinase that activates estrogen receptor α (ERα) and upregulates the transcription of the mitochondrial regulator, NRF1 and the IMS protease Omi (HTRA2) alleviating stress (Papa and Germain, 2011). Collectively, these findings lead to an essential question of whether the UPR^mt^ in mammals and *C. elegans* act similarly, and identifying homologous transcription factors in higher metazoans will shed light on this.

## UPR^mt^ and immunity

With the role of mitochondria as an innate immune hub being established, and UPR^mt^ central to mitochondrial health and resilience, scientists have long been interested in studying its involvement in immunity. It has only recently been observed that UPR^mt^ supports host tolerance by regulating mitochondrial homeostasis and endorsing host resistance through the transcription of innate immunity genes (Liu et al., 2014; Pellegrino et al., 2014; Jeong et al., 2017).

Report suggested that bacterial toxins which target the mitochondria and host cell trigger UPR^mt^ to counter mitochondrial dysfunction and initiate innate immune responses in *C. elegans* (Pellegrino et al., 2014). Using the *C. elegans* model, Pellegrino et al., 2014 studied the relationship between UPR^mt^ and innate immunity, and observed that UPR^mt^ confers long-term protection against *Pseudomonas aeruginosa* by inducing the expression of innate immune genes (Pellegrino et al., 2014). The involvement of ATFS-1 in upregulation of antibacterial factor-related peptide 2 (Abf-2), caenacin (CNC-4), lysozyme, clec-4 has also been reported in *C. elegans* (Nargund et al., 2012; Pellegrino et al., 2014; Nargund et al., 2015; Sapkota et al., 2021; Li et al., 2022).

Although UPR^mt^ is not well studied in higher animals, its involvement in inducing immune response genes and improving fitness against pathogens is getting increasingly evident (Wang et al., 2018; Chamseddine et al., 2022). UPR^mt^ components such as ATF5/ATFS-1 and HSP60 are upregulated during pathogenic infection in reef-building coral and the whiteleg shrimp (Chen et al., 2016; Dimos et al., 2019). ATFS-1 induces the expression of antimicrobial peptides required for survival following *Vibrio alginolyticus* infection in whiteleg shrimp (Chen et al., 2016). Our recent study suggested that infection with *Aeromonas hydrophila*-induced UPR^mt^ triggered apoptosis of infected fish macrophages by enhancing expression of *dnm1l* gene which encodes DRP1 protein, therefore results in the fragmentation of the mitochondrial network; and in turn, mitochondrial fission reduced mitochondrial membrane potential (ΔΨ_m_) which prompted release of cyt *c* in the cytosol leading to the activation of caspase-9 facilitating clearance of the intracellular bacteria (Kumar et al., 2022a). A recent study by reported that UPR^mt^ plays a protective role during enteric pathogenic infection in mammalian host. ATF5-mediated UPR^mt^ activation protects the mice against *Salmonella enterica* subsp. *enterica* (Serovar *Typhimurium*) by promoting intestinal barrier function *via* stimulation of satiety response and averting excessive glycolytic flux, which in turn prevented microbial and microbial toxins’ infiltration into underlying tissues (Chamseddine et al., 2022). ATF5 prevented intestinal barrier dysfunction by promoting cholecystokinin/leptin-mediated satiety response (Chamseddine et al., 2022). The list of UPR^mt^-induced genes along with their function in regulating immunity has been depicted in (**Table 1**). Though it is not evident whether different pathogens employ distinct mechanisms to induce UPR^mt^, these preliminary findings suggest that UPR^mt^-mediated regulation of innate immune genes is conserved across metazoans.

**Table 1:** UPR^mt^-induced genes in *C. elegans* and vertebrates.

In *C. elegans*		
**Function**	**Gene**	**References**
Metabolism	*ldh-2* (Lactate dehydrogenase), *clk-1* (Coenzyme Q biosynthesis), *glna-1* (Glutaminase), *clec-4* (C-type lectin domain family member 4)	(Nargund et al., 2012; Pellegrino et al., 2014; Nargund et al., 2015)
Transcription factor	*skn-1* (bZIP transcription factor, Nrf2 ortholog)	(Nargund et al., 2012; Nargund et al., 2015)
Innate immunity	*lys-2* (lysozyme), *abf-2, cnc-4* (antimicrobial peptide), *clec-4* (C-type lectin domain family member 4)	(Nargund et al., 2012; Pellegrino et al., 2014; Sapkota et al., 2021)
Mitochondrial protein import	*tomm-20* (translocase of the outer membrane subunit), *timm-17/23* (translocase of the inner membrane subunit 17/23)	(Rainbolt et al., 2013; Nargund et al., 2012; Nargund et al., 2015)
Mitochondrial protein homeostasis	*ppgn-1* (Mitochondrial paraplegin AAA protease), *hsp-6* (Mitochondrial hsp70), *dnj-10* (Mitochondrial DNA J), *ymel-1* (Mitochondrial AAA protease) *clpp-1* (Caseinolytic protease proteolytic subunit)	(Haynes et al., 2007; Nargund et al., 2012; Nargund et al., 2015)
Mitochondrial dynamics	*drp-1* (Dynamin-related protein), *mff-2* (Mitochondrial fission factor-2)	(Nargund et al., 2012; Nargund et al., 2015)
In vertebrates		
**Function**	**Gene**	**References**
Innate immunity	HD-5 (Antimicrobial peptide)	(Fiorese et al., 2016)
Mitochondrial protein homeostasis	mtHSP70 (Mitochondrial chaperone), LON (Mitochondrial protease) CLPP (Caseinolytic protease proteolytic subunit)	(Fiorese et al., 2016)
Mitochondrial metabolism	ASNS (Asparagine synthetase)	(Fusakio et al., 2016)
Mitokine	FGF21 (Fibroblast growth factor)	(Fusakio et al., 2016)
Mitochondrial protein import	*timm-23* (translocase of the inner membrane subunit 23)	(Rainbolt et al., 2013)
	MFN1 (Mitofusin 1) MFN2 (Mitofusin 2) OPA1 (Optic atrophy 1)	(Wang et al., 2018)
Mitochondrial dynamics	DRP1 (Dynamin-related protein)	(Kumar et al., 2022a)

Stressed mitochondria release DAMPS in bacterial infection (Jabir et al., 2015; Kumar et al., 2022a), which induce inflammatory responses and are critically involved in the pathogenesis of various diseases. UPR^mt^ may be regarded as an additional intracellular sensing mechanism that helps perceive damage inflicted by bacterial pathogens targeting mitochondrial bauplan and functions, and initiating remedial pathways crucial for restoring mitochondrial function and atoning inflammatory responses to promote cellular survival. Thus, it can be suggested that UPR^mt^ acts as a conduit coupling host resistance and tolerance, safeguarding the host during infection.

## Subverting UPR^mt^ activation

Recent reports demonstrated that bacterial pathogen subvert UPR^mt^ using bacterial enzymes (Mahmud et al., 2020) and targeting host factors (Deng et al., 2019) which regulate UPR^mt^ activation. For instance, Acyl-CoA dehydrogenase (FadE2) of *P. aeruginosa* possesses substrate specificity for the catabolites produced during the breakdown of the branched chain amino acids (valine and leucine), i.e., isobutyryl CoA and isovaleryl CoA respectively. During infection, bacterial FadE2 limits the availability of such catabolites for *C. elegans* through an unknown mechanism, which subsequently hinders in the activation of the UPR^mt^ (Mahmud et al., 2020). FadE2 impairs several energy pathways such as glycolysis, β-oxidation, and amino acid metabolism in *C. elegans*, all of which culminate with the TCA cycle. Consequently, loss of FadE2 in *P. aeruginosa*, results in restoration of energy metabolism due to availability of the catabolites in *C. elegans* and the ability to activate UPR^mt^ through a mechanism that is yet unknown. However, the exact molecular underpinnings of how FadE2 affects UPR^mt^ activity during infection *via* changes in valine or leucine levels is currently not resolved. Recently, it was shown that the *C. elegans* bZIP transcription factor, ZIP-3 is involved with the repression of the UPR^mt^ during *P. aeruginosa* infection (Deng et al., 2019). During infection, *P. aeruginosa* exploits the regulative activities of this transcription factor for manoeuvring UPRmt activation. Although, the mechanism of how *P. aeruginosa* manipulates ZIP-3 are unclear. The overlap observed between the genes negatively regulated by FadE2 and ZIP-3 strongly implicates some link between ZIP-3 and FadE2 in regulating UPR^mt^. *P. aeruginosa* negatively impacts both host energy metabolism and the activation of UPR^mt^ by outcompeting the host for specific nutrients. However, the molecular mechanisms linking these catabolites to host energy pathways and mitochondrial stress signaling are still unresolved, and therefore will be an exciting area of future research.

## Mitochondrial dynamics

Mitochondria are highly mobile organelles due to two significant processes termed mitochondrial dynamics: fission and fusion (Shaw and Nunnari, 2002; Chen and Chan, 2004). The term ‘mitochondrial dynamics’ refers to variations in shape, size, and localization of mitochondria and the system which controls these processes (Liesa and Shirihai, 2016). Besides assisting in various cellular functions such as oxidative phosphorylation, mitochondrial segregation into daughter cells at the time of cell division, propagation of intra-mitochondrial Ca^2+^ signal, and refurbishing of defective mitochondria (Chen and Chan, 2005; Twig et al., 2008; Westermann, 2010), the fission-fusion cycle also contributes in aiding the organelle to adapt to different conditions of stress (Westermann, 2012; Youle and van der Bliek, 2012; Ren et al., 2020). Mitochondrial dynamics also influence the kinetics of biochemical reactions, mitochondrial inner membrane topology, and protein super-complex assembly in the mitochondrial cristae (Lizana et al., 2008; Cogliati et al., 2013; Mannella et al., 2013). Most importantly, it has been reported that disruption of mitochondrial dynamics undermines their function and causes several human diseases (Suárez-Rivero et al., 2016; Chan, 2020).

## Fission and fusion: the mitochondrial ballet

**Figure 2:**
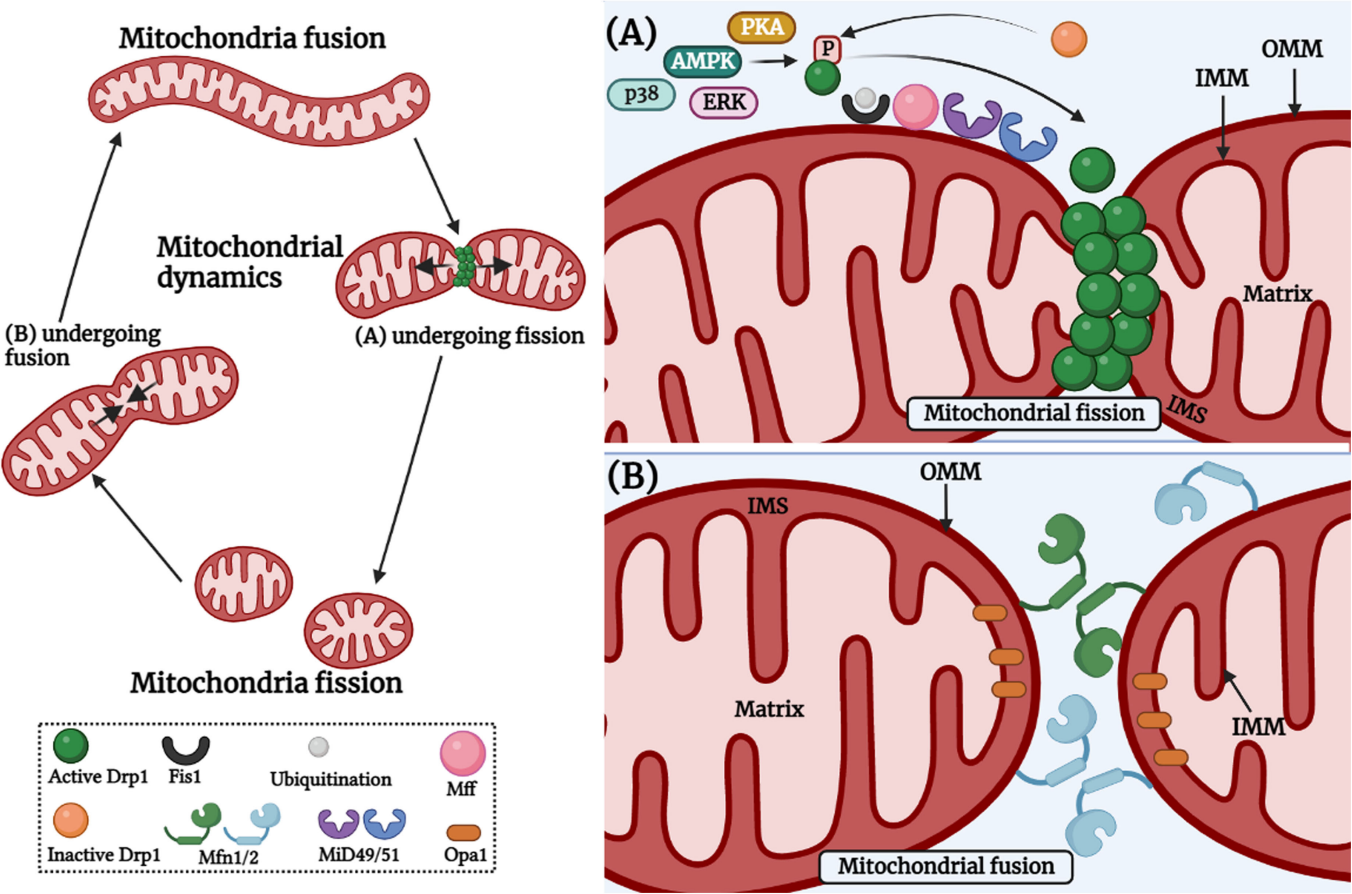
Mitochondrial fission and fusion cycle. Mitochondria are constantly dynamic cellular organelles and morphology of mitochondrial network keeps on changing. Mitochondrial fission involves the contact between endoplasmic reticulum and mitochondria, and the constriction occurs *via* actin cytoskeleton proteins. **(A)** Mitochondrial fission is controlled by post-translational modifications of Drp1, which includes S-nitrosylation, phosphorylation, SUMOylation, O-GlcNAcylation, and ubiquitination in response to various cellular stimuli *via* PKA, AMPK, ERK, p38 etc. After constriction, DRP1 is recruited to the fission site at the mitochondrial outer surface and interacts with adaptor proteins such as FIS1, MFF, MiD49 and MiD51 on the OMM (Right upper panel). **(B)** Mitochondrial fusion involves fusion of the OMM which occurs *via* the interaction of MFN1 and MFN2 between two individual mitochondria. Then, fusion of the IMM occurs with the help of OPA1 GTPase which is present at the IMM (Right lower panel). The image was created with the help of BioRender.com.

A group of classical and distant dynamin-related GTPases regulates the fusion-fission process. Mitochondrial fusion is a two-step process highlighted by the union of the outer mitochondrial membrane (OMM) followed by the union of the inner mitochondrial membrane (IMM) of two originally distinct mitochondrion. This process is controlled by IMM protein, optic atrophy 1 (OPA1), and OMM proteins-mitofusin 1 and mitofusin 2 (MFN1 and MFN2). The trans-interaction between MFNs on opposing OMMs involves continuous hydrolysis of GTP, whereas the IMM fusion depends on OPA1 assembly stimulating GTPase activity and membrane tubulation (Gao and Hu, 2021) (**Figure 2**). It has been speculated that OPA1-mediated inner membrane fusion may occur only after receiving a signal from active mitofusins on the outer membrane, thus coordinating the fusion process (Hoppins, 2014). In the case of mitochondrial fission, a classical dynamin-related protein, termed dynamin-related protein 1 (DRP1) in mammals, is the master regulator of mitochondrial fission machinery (Westermann, 2010). It is a soluble protein containing an N-terminal GTPase, a middle domain, and a c-terminal GTPase effector domain involved in self-assembly. The fission cycle starts with the involvement of two partner proteins, mitochondrial fission 1 (FIS1) and mitochondrial fission factor (MFF) (Losón et al., 2013; Kraus et al., 2021). First, FIS1, an OMM-anchored protein, recruits MFF from the cytosol to the OMM, leading to the nucleation of GTP-bound DRP1 oligomers on the OMM (Palmer et al., 2011; Losón et al., 2013). Next, in this line, DRP1-GTP oligomers form spirals that are eventually wrapped around the organelle and sever the mitochondrial membranes following GTP hydrolysis (Mears et al., 2011) (**Figure 2**). Mitochondrial fusion and fission processes are antagonistic. The right balance between these two must be achieved by tightly controlling the rate of fusion and fission. This regulation is a prerequisite for the maintenance of mitochondrial morphology or for adapting to ever-changing physiological conditions.

## Mitochondrial dynamics in immunity

In the past decade, numerous studies have revealed the importance of mitochondrial metabolism in immunity (Bahat et al., 2021; Timblin et al., 2021) and mitochondrial dynamics are essential for immune responses mediated by various cell types (Mishra and Chan, 2016; Wai and Langer, 2016) and interconnected with MQC (Ježek et al., 2018) such as UPR^mt^. The disruption of mitochondrial dynamics has detrimental consequences for mitochondrial and cellular homeostasis, and this leads to the activation of the UPR^mt^ (Kim and Sieburth, 2018; Rolland et al., 2019; Haeussler et al., 2020; Haeussler et al., 2021). Loss of intestinal and neuronal *fzo-1* (encodes for mitofusin) in *C. elegans* induces fragmentation of the mitochondrial network, reduces mitochondrial membrane potential (Haeussler et al., 2020; Chen et al., 2021) which in turn triggers UPR^mt^ in intestine (Chen et al., 2021). Chen et al., 2021 further reported that silencing of neuronal *fzo-1* triggers non-autonomous UPR^mt^ *via* neuromodulators such as tyramine, and neurotransmitters such as serotonin (Chen et al., 2021). A recent report highlighted the role of neuronal signals in non-autonomous UPR^mt^ regulation. *unc-17* (vesicular acetylcholine transporter), *eat-4* (vesicular glutamate transporter), *tdc-1* (tyrosine decarboxylase), *tph-1* (tryptophan hydroxylase) mutations suppressed UPR^mt^ activation which clearly implicated that acetylcholine, glutamate, tyramine, and serotonin are essential in communicating mitochondrial stress between neurons and intestine. The interruption in the activity of RIM and RIC significantly reduced non-autonomous UPR^mt^ activation, and tyramine plays a crucial role in propagating non-autonomous UPR^mt^ *via* TYRA-3 receptor. And in addition to this, perturbation of neuronal mitochondrial dynamics activated UPR^mt^ without any involvement of mitochondrial respiration (Chen et al., 2021) which clearly implicates direct involvement of mitochondrial fragmentation in UPR^mt^ activation, and activation of UPR^mt^ in *C. elegans* confers protection against *P. aeruginosa* infection (Pellegrino et al., 2014).

In *C. elegans*, knockdown of *fzo-1*, *eat-3* which regulate mitochondrial fusion or *drp-1* which regulates mitochondrial fission induces UPR^mt^ (Kim and Sieburth, 2018; Zhang et al., 2018). Mitochondrial dynamics affects levels of triacylglycerols, and *fzo-1* mutants exhibited significant reduction in the number of short-chain length triacylglycerols, and increase in unsaturated triacylglycerols (Haeussler et al., 2020) implicating alteration in the contact sites between mitochondria and triacylglycerols. Disruption in mitochondrial fusion affects transfer of fatty acid from lipid droplets to mitochondria in mammalian cells, leading to heterogeneous distribution of fatty acid among mitochondria (Rambold et al., 2015), and loss of *fzo-1* or *drp-1* results in disruption of the contact sites between lipid droplets and mitochondria (Haeussler et al., 2020); and such alterations induces metabolic changes. Autophagy suppresses UPR^mt^. And recent report highlighted the involvement of mitochondrial dynamics in autophagy-mediated UPR^mt^ regulation (Haeussler et al., 2020). Autophagy regulates metabolism of fatty acids, it triggers breakdown of triacylglycerols, and thus ensures a continuous supply of fatty acids to mitochondria for β-oxidation (Singh et al., 2009). And thus, autophagy reverts such changes in the levels of triacylglycerols in *fzo-1* mutants (Haeussler et al., 2020). Hence, autophagy triggers enhanced breakdown of triacylglycerols in mutants with defects in mitochondrial dynamics, that are subsequently used to fuel mitochondrial metabolism, and thereby increases mitochondrial membrane potential leading to the suppression of UPR^mt^. Decrease in mitochondrial membrane potential has recently been shown to be a prerequisite signal for UPR^mt^ induction (Rolland et al., 2019). The first evidence that autophagy can affect UPR^mt^ was the finding by Haynes et al. that knock-down of rheb-1, a known positive regulator of TOR (Honjoh et al., 2009), and autophagy is dependent on ATFS-1 (Nargund et al., 2012; Guo et al., 2014) and induction of autophagy leads to improved mitochondrial function by affecting lipid metabolism and ameliorating cellular homeostasis, thereby suppressing UPR^mt^ in mutants with defects in mitochondrial dynamics. These reports clearly demonstrate the relationship between mitochondrial dynamics and UPR^mt^. Further, the below mentioned section mainly discusses the role of mitochondrial dynamics in immunity and host-pathogen infections.

### Mitochondrial dynamics in innate immunity

Innate immunity is the first line of defence against invading pathogens. It is evolutionarily conserved and shows little variation while responding to pathogens. Neutrophils are essential constituents of the innate immune system. These cells are recruited earliest to the sites of infection and respond by releasing ROS, proinflammatory cytokines, and neutrophil extracellular traps (NETs) (Papayannopoulos, 2018; Schultz et al., 2022). It was observed that silencing of OPA reduced ATP production and NET formation, implicating mitochondrial fission’s role in the process (Cervantes-Silva et al., 2021). Monocytes are phagocytic cells that can differentiate into macrophages and dendritic cells. Under pathogenic stress, circulating blood monocytes are recruited to the sites of inflammation and differentiate into macrophages (Shi and Pamer, 2011). The metabolic status of monocytes alters in response to the environmental stimuli, and the cellular metabolism plays a crucial role in monocyte activation in which mitochondria is a major central player in regulating cellular metabolism. Hence, mitochondrial dynamics is closely associated with monocyte-macrophage differentiation. And mitochondrial dynamics is reported to play a critical role in monocyte-macrophage differentiation (Rambold and Pearce, 2018). Stimulation of human PBMCs by TLR4 agonist i.e., LPS induced differentiation of monocytes into macrophages (Krutzik et al., 2005). The size of the mitochondrial network and mitochondrial footprints, i.e., mitochondrial mass increases in BMDMs as compared to the PBMCs which showed tubular mitochondria (Li et al., 2020), which clearly suggested mitochondria of monocytes undergo biosynthesis and elongation during monocyte-macrophage differentiation. Furthermore, PBMCs mainly relies on the basal level of OXPHOS and glycolysis (Chacko et al., 2013), whereas, BMDMs relies on enhanced OXPHOS activity (Na et al., 2015), and the elongation of mitochondrial network elevates OXPHOS activity (Yao et al., 2019). Pre-treatment of CD14^+^ human monocytes with LPS triggers their activation which involved reduced expression of MTFP1 (Mitochondrial Fission Process 1), which in turn increase mitochondrial fusion (Duroux-Richard et al., 2016). Similarly, elongation of mitochondrial network is also reported during monocyte to macrophage differentiation in murine cells (Li et al., 2020). TLR-4 senses LPS of Gram-negative bacteria, and TLR-4-LPS axis results in marked inhibition in the expression of mitochondrial fission machinery with a concordant increase in mitochondrial fusion (Duroux-Richard et al., 2016; Cervantes-Silva et al., 2021). However, reports also suggest TLR signaling alters mitochondrial morphology initiating a switch from fusion to fission (Hoppins, 2014; Kumar et al., 2022a). Though contradictory, these findings are proof of the involvement of TLRs in mitochondrial dynamics. Notably, the TLR family contains several members and isoforms thereof. Given the evolutionarily conserved nature of TLR signaling it will be interesting to determine how mitochondria perceive TLR signaling and whether the molecular cross-talk between mitochondria and different TLRs is the same. Both mitochondria and TLR represent keystones of innate immunity, future research addressing how mitochondrial dynamics harness TLR signaling and vice versa will help in therapeutics for innate immune disorders.

Macrophages are terminally differentiated phagocytes broadly classified into pro-inflammatory M1 and anti-inflammatory M2 sub-types (Cervantes-Silva et al., 2021). The M1 phenotype exhibits a fragmented mitochondrial network, and M2 phenotype shows elongated and fused mitochondria (Hinshaw et al., 2021). LPS stimulation triggers the development of the M1 subtype by inhibiting the expression of fusion molecules (MFN1 and MFN2) and phosphorylation of Drp-1 (Ser637) critical for triggering the fission pathway (Gao et al., 2017). Mitochondrial dynamics are crucial in regulating inflammasome activation in macrophages which help host cells against microbial infections. It has been shown by several groups that the expression of mitochondrial fusion proteins and defective mitochondrial fission favour inflammasome formation (Ichinohe et al., 2013; Park et al., 2015). Mast cells are involved in hypersensitivity reactions and innate immunity to several pathogens. They recognize ligands through FcϵRI/II receptors and act by releasing secretory granules, which induce diverse effects depending on the nature of the ligand and anatomical locations. Inhibiting DRP1 activity negatively affects mitochondrial translocation and granule exocytosis suggesting the importance of mitochondrial dynamics in mast cell functioning (Zhang et al., 2011).

### Mitochondrial dynamics in adaptive immunity

Mitochondrial dynamics have been linked with several aspects of B- and T-cell functioning. Studies by several groups have suggested an intimate relationship between mitochondrial dynamics and immunological synapse formation and regulation (Campello and Scorrano, 2010; Quintana and Hoth, 2012). Inhibiting the expression of Drp1 was found to have little effect on T-cell differentiation but hindered T-cell migration and activation following antigenic challenge (Simula et al., 2018). The naive T-cells differentiate into effector (T_E_) and memory (T_M_) subtypes upon antigenic exposure. While the naive T-cells have small fragmented mitochondria and are essentially dependent on mitochondrial OXPHOS for energy requirements, T_E_ cells undergo a quantum increase in mitochondrial mass *via* Drp-1 mediated fission and acquire their energy quotient by aerobic glycolysis (Buck et al., 2016). Drp-1 mediated fission also aids in generating mtROS (Gao et al., 2017) which in turn influences the activation of nuclear factor of activated T cells (NFAT) and subsequent IL-2 production, essential for the functioning of T_E_ cells (Sena et al., 2013). Prolonged mtROS generation is deleterious for cellular health, and how T_E_ cells regulate the Drp-1/mtROS cascade, thereby maintaining a delicate balance between clonal activation and clonal deletion, merits future investigations. Alteration in mitochondrial mass and ensuing morphological changes are reported to play a critical role in the development of T_M_ cells. The T_M_ cells have hyper-fused mitochondria and depend on fatty acids for energetics and recall responses. Inhibition of OPA1 expression reduced T_M_ cell survival, and further reported that pharmacological and genetic ablation/silencing of Drp-1 or endorsing OPA1 expression tilts the balance in favour of T_M_ cell development (Buck et al., 2016). Importantly, T_M_ cells are long-lived and require a low energy budget which also implicates the role of mitochondrial fusion in their longevity. These findings suggest mitochondrial dynamics is essential in shaping different aspects of T-cell biology, but how the fine-tuning between two disparate events, fission, and fusion, is achieved needs to be better understood.

Right from inception in the bone marrow, B-cells undergo cycles of differentiation and activation, each stage posing distinct metabolic demands. Like T-cells, activated B-cells have a higher energy demand, and depend more on anabolic metabolism for their energy requirements than naive B-cells (Sandoval et al., 2018). The replacement of elongated mitochondria in naive B-cells with numerous rounded mitochondria following activation suggests the involvement of mitochondrial fission in B-cell activation (Waters et al., 2018). Though these early findings reflect the intimate relationship between the two molecular events, further studies are needed to cement the role of mitochondrial dynamics on B-cell development, differentiation, and functioning.

### Mitochondrial dynamics at the host-pathogen interface

**Figure 3:**
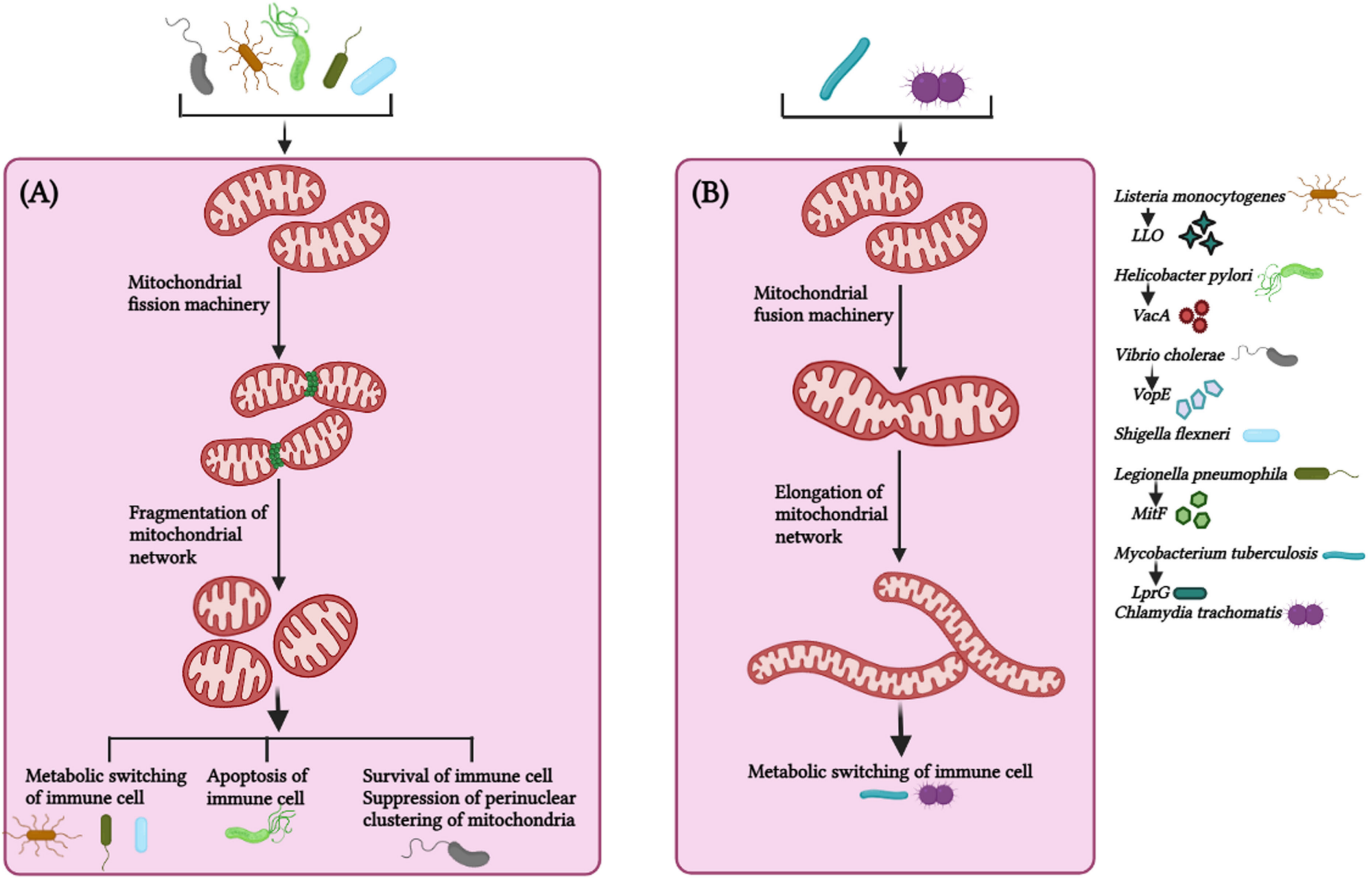
Mitochondrial dynamics in host immunity against bacterial infection. **(A)** Bacteria such as *Listeria monocytogenes*, *Helicobacter pylori*, *Vibro cholerae*, *Shigella flexneri*, *Legionella pneumophila* triggers fragmentation of the mitochondrial network *via* virulence factors (LLO – Listeriolysin, VacA – Vacuolating cytotoxin A, VopE – mitochondrial targeting T3SS effector protein, MitF – T4SS effector protein, respectively and effector protein in case of *Shigella flexneri* is not known) to maintain a favorable microenvironment for pathogen survival and thus promote bacterial survival, persistence and metabolic switching of the immune cells. **(B)** Bacteria such as *Mycobacterium tuberculosis* and *Chlamydia trachomatis* circumvent host immune response by manipulating the mitochondrial dynamics and maintains elongated network of mitochondria at initial phase of infection for their survival. Alteration in mitochondrial fission (by *L. monocytogenes, H. pylori, V. cholerae, S. flexneri, L. pneumophila*) and mitochondrial fusion (*M. tuberculosis, C. trachomatis*) suppress bacterial replication and facilitate bacterial clearance from the cell. *M. tuberculosis* utilize LprG (Lipoarabinomannan carrier protein) for mitochondrial fusion. The image was created with the help of BioRender.com.

Mitochondrial dynamics, the key to mitochondrial health, has been targeted by numerous pathogens for survival and causing infections. Pathogenic bacteria have been shown to modulate mitochondrial dynamics to create a niche ideal for intracellular replication, immune evasion, and persistence (Khan et al., 2020; Tiku et al., 2020). The role of mitochondrial dynamics on host immunity has been studied in detail in *Listeria monocytogenes* pathogenesis. *L. monocytogenes* elicits short-term mitochondrial fission and reduction in energy production using the pore-forming toxin listeriolysin O (LLO) (**Figure 3A**). Mitochondrial respiration restricts the growth of this intracellular pathogen, and it survives by sophisticated manipulation of mitochondrial dynamics (Stavru et al., 2011). During infection, it hijacks the mitochondrial metabolism machinery enforcing the expression of receptors that aid pathogen entry inside cells, suggestive of novel manoeuvring of mitochondrial function by the pathogen (Spier et al., 2021). Additionally, while pathogenic *L. monocytogenes* fails to infect cells with fragmented mitochondria, the infection was more prevalent in cells with fused mitochondria (Stavru et al., 2011), suggesting the positive correlation between infectibility and *L. monocytogenes*-induced mitochondrial fission is critical for pathogenesis induced by the bacterium. Interestingly, *L. monocytogenes*-induced mitochondrial fission is transient and occurs in the early phase of infection (Stavru et al., 2011). Selectively impairing mitochondrial dynamics at the initial stages provides *L. monocytogenes* a window for successful infection and ensures that the infected cell remains free of DAMPS released by damaged mitochondria, which otherwise induces pro-inflammatory responses counterproductive to the pathogen. How the bacteria sense the extent of mitochondrial damage and precisely manoeuvre the mito-repair machinery need to be studied. Recently, it was reported that Mic10, a critical component of the mitochondrial contact site and cristae organizing system (MICOS) complex, is responsible for *L. monocytogenes*-induced mitochondrial fission (Carvalho et al., 2020), suggestive of novel mechanisms used by pathogens to circumvent host immunity and it is crucial to know whether other pore-forming bacteria use similar mechanisms to establish infections.

*Helicobactor pylori* cause persistent gastric infections leading to peptic ulcers and stomach cancer. The bacteria release vacuolating cytotoxin VacA, which activates Drp1-mediated fragmentation of the mitochondrial network culminating in apoptosis of infected cells and pathogen survival (Jain et al., 2011) (**Figure 3A**). Inhibiting Drp-1 attenuated the expression of pro-apoptotic Bax and cytochrome C besides helping in pathogen clearance suggesting the cross-talk between mitochondrial dynamics and apoptosis critical for *H. pylori* pathogenesis (Jain et al., 2011).

The non-invasive pathogen *Vibrio cholerae* colonizes the small intestine and produces cholera toxin, causing severe secretory diarrhoea. Miro (mitochondrial Rho GTPases) is a mitochondrial outer membrane protein that regulates mitochondrial dynamics by interacting with MFN1, MFN2, OPA1, DRP1, and FIS1 (Chan et al., 2006; Westermann, 2010; Panchal and Tiwari, 2021). *V. cholerae* triggers fragmentation of the mitochondrial network and restricts perinuclear clustering of the mitochondrial network, which is critical for mitochondria-mediated immune responses (Suzuki et al., 2014) (**Figure 3A**). The overexpression of EFm mutants of Miro or Mfn 1 mutant (loss-of-function GTPase in activity) significantly occluded NF-κB activation and the clustering of the perinuclear mitochondrial network during infection (Suzuki et al., 2014), implicating the involvement of mitochondrial dynamics in regulating *V. cholerae* pathogenesis. *Shigella flexneri* utilizes mitochondrial dynamics for successful infection (**Figure 3A**). Silencing DRP1 expression revokes mitochondrial fragmentation, reduces the bacterial load, and prevents the cellular spread of *S. flexneri* (Lum and Morona, 2014).

*Legionella pneumophila* is a gram-negative bacterium responsible for Legionnaires’ disease. Inhibition of mitochondrial fragmentation or promotion of mitochondrial fusion decreases *L. pneumophila* replication (Escoll et al., 2017). *L. pneumophila*-induced alterations in mitochondrial dynamics *via* MitF allow the repurposing of the metabolism in macrophages to impair oxidative phosphorylation and activate the glycolytic cycle (**Figure 3A**). This metabolic shift of the infected macrophages strikingly resembles the Warburg effect, which is exploited by *L. pneumophila* for replication (Escoll and Buchrieser, 2018). Based on these observations, the inhibition of mitochondrial fission halts the onset of Warburg effect and restrains *L. pneumophila* infection in macrophages.

A Warburg effect-like scenario is observed with *Mycobacterium tuberculosis* infection, but this is quintessential for host cells to clear the intracellular pathogen (Aguilar-López et al., 2019) (**Figure 3B**). The probable mechanism behind this *M. tuberculosis*-induced transient altered mitochondrial dynamics is that mitochondrial fragmentation of the mitochondrial network at early stages provides a suitable micro-environment for effective infection and survival by impairing the metabolism of macrophages. In contrast, mitochondrial fragmentation is attenuated at later stages to reduce the damage to the infected macrophages, thus enabling persistent infection.

Interestingly, some pathogens invoke mitochondrial fusion for survival and spread. *Chlamydia trachomatis* is responsible for STD in males and females. The bacteria lack several key enzymes for metabolic activities and depend on the host for energy and survival. *Chlamydia* increases mitochondrial fusion by inhibiting DRP1 activation and maintains the mitochondrial network in the host for meeting energy requirements and its proliferation (Chowdhury et al., 2017; Kurihara et al., 2019) (**Figure 3B**). It was noted that *C. trachomatis* induces elongation of mitochondria in the early phase of infection but augments mitochondrial fragmentation during the later phases (Kurihara et al., 2019; Rother et al., 2019), the reason being that *C. trachomatis* requires ATP from the host cells for enhancing intracellular growth, and elongation of mitochondrial network *via* cAMP-mediated phosphorylation of Drp1-S637 promote ATP synthesis at the initial stages of infection, and later on, *C. trachomatis* shifts the metabolism from mitochondrial respiration to aerobic glycolysis in order to meet up the high metabolic demands of rapidly growing intracellular bacteria inside the cells. The Warburg effect supports the rapid proliferation of the intracellular bacteria by increasing cellular biomass, in which mitochondrial respiration is decreased and aerobic glycolysis is enhanced, therefore, *C. trachomatis* induces mitochondrial fission at the later stages of infection, such strategies by intracellular bacteria implicates the requirement of distinct morphology of mitochondrial network at different stages of infection. **Table 2** summarizes the alteration of mitochondrial dynamics in response to diverse pathogenic infection (**Table 2**).

**Table 2:** Alteration in mitochondrial dynamics in response to various bacteria.

Bacteria	Affected protein(s)	Effector bacterial protein(s) involved	Consequent mitochondrial morphology	Effect on host and/or pathogen	References
*Acinetobacter baumannii*	DRP1	OmpA	Induces mitochondrial fragmentation	Induces host cell death	(Tiku et al., 2021)
*Aeromonas hydrophila*	DRP1	?	Induces mitochondrial fragmentation	Induces pathogen clearance	(Kumar et al., 2022a)
*Chlamydia trachomatis*	DRP1	?	Induces mitochondrial elongation	Improved pathogen survival	(Chowdhury et al., 2017; Kurihara et al., 2019)
*Helicobacter pylori*	DRP1	VacA	Induces mitochondrial fission	Induces host cell apoptosis	(Jain et al., 2011)
*Legionella pneumophila*	DRP1	MitF	Induces mitochondrial fission	Improved pathogen survival	(Escoll et al., 2017)
*Listeria monocytogenes*	MICOS10	LLO	Induces mitochondrial fission	Improved pathogen survival	(Stavru et al., 2011; Carvalho et al., 2020)
*Mycobacterium tuberculosis*	MFN2	ESAT6, LrpG	Induces mitochondrial hyperfusion	Induces inflammasome activation and IL-1β secretion	(Aguilar-López et al., 2019; Xu et al., 2020)
*Shigella flexneri*	DRP1	?	Induces mitochondrial fission	Induces non-apoptotic host cell death	(Lum and Morona, 2014)
*Vibrio cholerae*	Miro1 and Miro2	VopE	Prevents Mfn1-induced mitochondrial fusion	Inhibits host inflammatory responses	(Suzuki et al., 2014)

?, Not yet identified.

Recently, MFN2 has been reported as a master regulator of the immune response against bacterial infection (Tur et al., 2020). Out of all three mitochondrial fusion proteins, only MFN2 is highly expressed in macrophages predominantly upon immune stress signals such as pro-inflammatory activators (e.g., TLR ligands) (Lloberas et al., 2020), which also suggests that despite being highly homologous, MFN2 differs from MFN1 in certain functions. For example, mtROS production was impaired in MFN2-depleted macrophages, while depletion of MFN1 did not alter mtROS production in response to LPS endotoxemia (Tur et al., 2020). As expected, MFN2-depleted macrophages show highly fragmented mitochondria and a drastic reduction in mitochondrial membrane potential. MFN2-deficient macrophages cannot enhance their respiration rate in response to a metabolic change induced due to LPS endotoxemia (Tur et al., 2020). The depletion of MFN2 in macrophages affects phagocytic activity, as MFN2^-/–^macrophages showed a reduction in uptake of *Aeromonas hydrophila*, *Staphylococcus aureus*, and *E. coli* (Tur et al., 2020), which highlights the importance of this mitochondrial fusion protein in enhancing phagocytic activity of macrophages. Mice deficient in MFN2 are more susceptible to intracellular bacterial pathogens such as *Mycobacterium tuberculosis* and *Listeria monocytogenes*. Furthermore, the survival rate of MFN2^-/-^ mice is strongly reduced in LPS-induced endotoxemia (Tur et al., 2020).

Thus, it can be concluded that the imbalance in mitochondrial dynamics results in diametrically opposite pro-host and pro-microbial outcomes. Although some progress has been made in this field dealing with the perturbation of mitochondrial dynamics at the host-pathogen interface, there are still many unresolved questions, including the mechanism of interaction between mitochondrial dynamic proteins and bacterial virulence factors. Understanding these aspects will improve our understanding of the involvement of mitochondria in regulating bacterial pathogenesis. The functional correlation between mitochondrial dynamics and bacterial pathogenesis should be further elucidated to develop new antibacterial treatment strategies and efficient therapeutics.

## Conclusion

**Figure 4:**
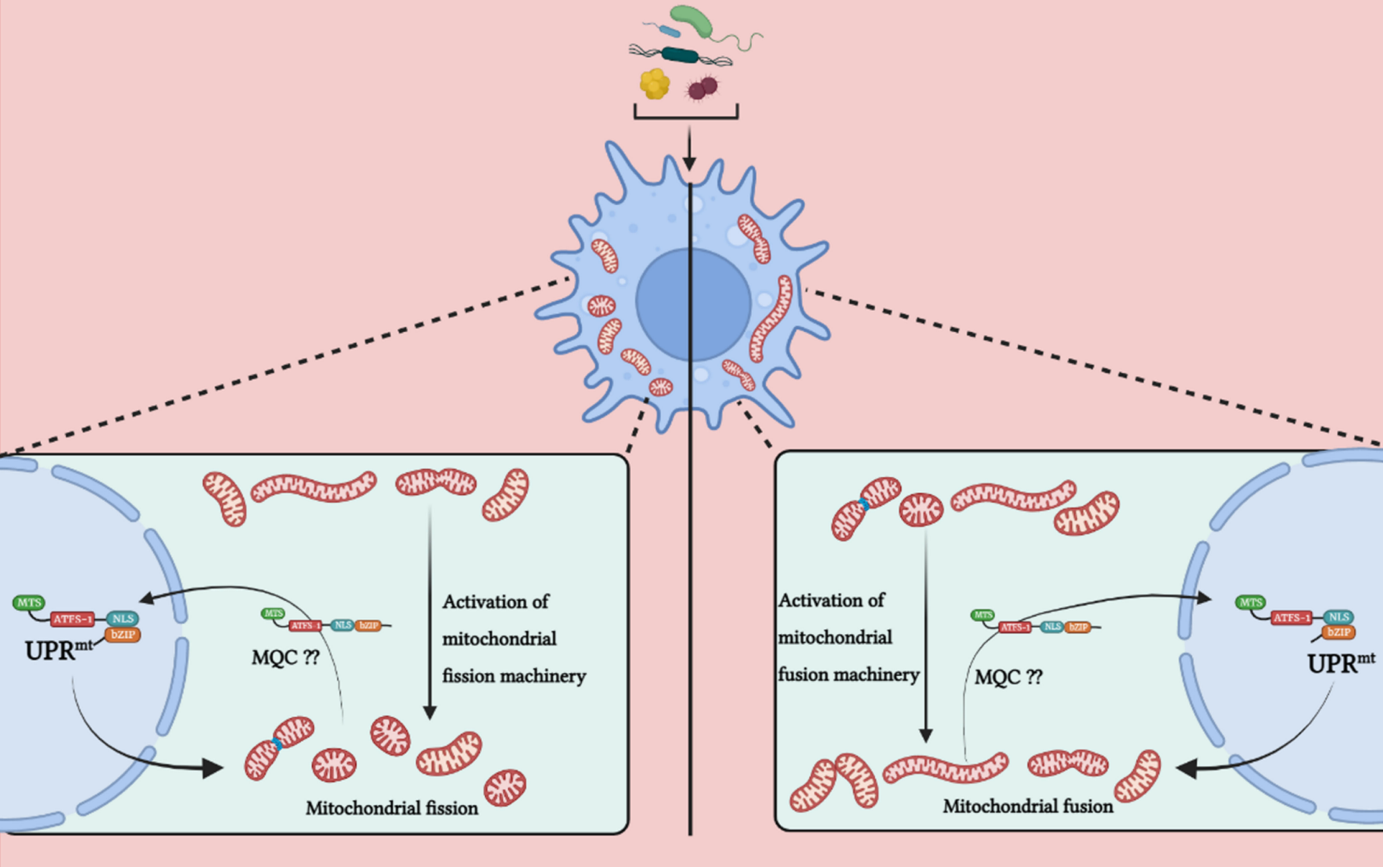
The involvement of mitochondrial fission, fusion and UPR^mt^ in host immunity.

Mitochondria acts as a highly versatile player in governing different aspects in immune cells for mounting an efficient host response against bacterial infections, thus, it is understandable that variety of intracellular bacteria have evolved explicit strategies to hijack mitochondrial functions in order to create a viable niche for themselves. Likewise, extracellular bacteria have also developed numerous ways of targeting mitochondria to induce cell death to avail themselves with nutrients. Downstream of a bacterial infection, it is fascinating that a plethora of immune responses, be it against bacteria or bacterial products, are strongly impacted by mitochondrial dynamics and UPR^mt^ (**Figure 4**). However, the investigations of these two functional aspects of mitochondria in immune response just started to unravel recently therefore, some key queries (see - In need of answers) still need a systematic comprehensive investigation. Thus, it will be exhilarating to extrapolate this current understanding and explore into how these fundamental mechanisms governed by mitochondria can be translated into active therapeutics to foster immunity against bacterial pathogens, and/or to keep unconcealed immune responses under control in the case of inflammatory disorders induced by bacterial infection.

**In need of answers**

- What sort of peptides are effluxed by HAF-1 into the cytosol that halts ATFS-1 translocation into the mitochondria?

 ◦ Specifically which peptides are involved in this process, need to be investigated.
 ◦ Do these peptides change the conformation of ATFS-1 in cytosol, exposing NLS region; and if yes, what are the underlying mechanisms?

- Why does the manipulation of mitochondrial dynamics vary differentially in immune cells by different species of bacterial pathogens?

 ◦ Is this variation in mitochondrial dynamics in bacterial infection depends upon variety of bacterial virulence factors, or host factors, or interaction of these factors decide the state of mitochondrial dynamics?

- Does the targeting of mitochondrial processes only *via* therapeutics reinforce immune response against bacterial infection and consequently, restrain their replication and spreading?

## Author contributions

MK, SS, and SM designed the theme of the manuscript. MK and SS conducted the literature search and wrote the manuscript. MK and SS drew the schematic diagram and tables. SM critically reviewed and edited the final version of the manuscript. All authors read and approved the final manuscript.
